# Through the lens of phosphorus: The legacy of Andrew Sharpley

**DOI:** 10.1002/jeq2.70032

**Published:** 2025-05-21

**Authors:** Peter J. A. Kleinman, Don Flaten, Deanna Osmond, Helen Jarvie, Richard McDowell, Zachary Simpson, Joshua Mott

**Affiliations:** ^1^ USDA‐ARS Center for Agricultural Resources Research Soil Management and Sugar Beet Research Unit Fort Collins Colorado USA; ^2^ Faculty of Agricultural and Food Sciences University of Manitoba Winnipeg Manitoba Canada; ^3^ Department of Crop and Soil Sciences North Carolina State University Raleigh North Carolina USA; ^4^ Department of Geography and Environmental Management University of Waterloo Waterloo Ontario Canada; ^5^ AgResearch New Zealand Lincoln New Zealand; ^6^ Department of Soil and Physical Sciences Lincoln University Lincoln New Zealand; ^7^ USDA‐ARS Sustainable Water Management Research Unit Stoneville Mississippi USA

## Abstract

The pursuit of sustainable phosphorus (P) management represents a long‐standing challenge in agricultural arenas, with far‐reaching implications for the environment and societal development. Few scholars are as synonymous with P science as Andrew Sharpley. Renowned over his 44‐year professional career for providing foundational insights into the fate and management of agricultural P, Sharpley also became the central figure in organizing responses to concerns over the contribution of non‐point source P pollution to eutrophication. As a global authority, Sharpley led teams that crafted scientific consensus for use by managers and policymakers in the face of complexity and uncertainty. His leadership offers lessons to those continuing in his footsteps and a model for all who desire to achieve scientific impact. This special collection of papers celebrates the perspectives, contributions, and legacy of Andrew Sharpley.

## Core Ideas

- Few scholars are as synonymous with phosphorus science as Andrew Sharpley.
- We provide a biography of Sharpley's career.
- Sharpley's efforts provide a model for how to achieve scientific consensus and impact.

**Abbreviation**
EPIC: Environmental Policy Integrated Climate

## 1 INTRODUCTION

In the realm of phosphorus (P) science, Andrew Sharpley's influence is unparalleled. Over 44 years, Sharpley's dedication to exploring the many dimensions of agricultural P established many important landmarks in the field, and his science continues to frame research and practice across the globe. As described in this special collection of papers reflecting upon Sharpley's legacy, his contributions to environmental and agricultural science, nutrient and watershed management, strategic planning, and computational modeling influenced many others because of the clarity, creativity, utility, and scholarship that were hallmarks of Sharpley's work. Indeed, Sharpley's role in agricultural P science offers lessons to the modern scientist in (a) how to build bridges of consensus in the midst of uncertainty so that one's science translates into practice, (b) how to embrace scientific uncertainty and new ideas with integrity so that one's work continues to be relevant as knowledge relentlessly evolves, and (c) the importance of remaining humble and accessible to colleagues in the course of achieving global recognition.

## 2 BIOGRAPHY

Andrew Neville Sharpley grew up in a comfortable, working‐class home in urban Manchester, well into the reconstruction of Britain after World War 2. Curious, and with a keen interest in the natural world, Sharpley entered University College of North Wales in 1969 where he eventually majored in Soil Science and Biochemistry with a minor in Oceanography. It was during these studies in northern Wales—where land and sea can appear intertwined—that Sharpley was first introduced to agricultural science, a field that piqued his interest given its practical ties to natural systems, and a subject that provided meaning for a budding scientist wanting to contribute to a greater good.

It is noteworthy that Sharpley's own recollection of his professional trajectory is grounded in personal relationships, colored by descriptions of the personalities who were his professional influences. Recruited to graduate school at Massey University in New Zealand by the late Keith Syers, whose seminal work with Thomas Walker (New Zealand's first professor of soil science) is foundational to our understanding of P in pedogenesis (Walker & Syers, 1976), Sharpley pursued his graduate training in the agricultural hub of Palmerston North. Beginning in 1973, Sharpley was immediately introduced into the dynamic world of collaborative research. Within Syers’ program, Sharpley joined a team of ambitious young researchers pursuing a variety of scientific disciplines, all charged by Syers to develop a collective understanding of the fate and management of nutrients threatening New Zealand's pristine waterways.

It was through shared discussions in graduate school with labmates and his own empiricism that Sharpley first developed clarity around the interplay of P in the terrestrial environment with its fate in aquatic systems. During this time, Sharpley worked to elucidate the mechanisms of dissolved P mobilization (A. N. Sharpley et al., 1977) and P transport to and from tile drains (A. N. Sharpley & Syers, 1976), both subjects relevant today that have been repeatedly debated (or rediscovered) in other settings by those who appreciate Sharpley's foundational research. Sharpley's graduate research also taught him the importance of experiential knowledge and direct observation in field science. In an era before automatic samplers, Sharpley endured countless hours of wet, cold conditions to obtain grab samples from waterbodies during storm events (which, according to Sharpley, seemed to predominately occur between midnight and dawn hours!). This experience served as a right of passage, but, more importantly, provided the developing P scholar with direct observation of the hydrologic processes involved in mobilizing and moving P. According to Sharpley, such experiential learning, along with its associated suffering and “character‐building,” is unappreciated by many modern students.

**Core Ideas**

- Few scholars are as synonymous with phosphorus science as Andrew Sharpley.
- We provide a biography of Sharpley's career.
- Sharpley's efforts provide a model for how to achieve scientific consensus and impact.

After completing his Ph.D. in 1977, Sharpley remained briefly at Massey University as a postdoctoral scholar before moving across the Pacific Ocean to pursue a second postdoc in the United States at the University of California, Davis. Sharpley did not linger at Davis, as he was immediately recruited by Ron Menzel into USDA's Agricultural Research Service laboratory in Durant, OK. Here, he had the good fortune to work with Sam Smith and Laj Ahuja, who played important roles as an early‐career mentors. Smith reinforced Sharpley's emphasis on the practical implications of his research and encouraged Sharpley's explorations in new areas of science, while Ahuja helped Sharpley to discover theoretical, physical, and chemical interpretations of field observation that were critical to future runoff modeling efforts.

It was during his nearly 17 years with USDA in Oklahoma (1978–1995) that Sharpley's writing talents, comprehensive understanding of mechanisms controlling P fate and transport, and innovation in developing strategies for science and management achieved a high degree of recognition (e.g., A. N. Sharpley, 1985). During this time, Sharpley refined highly relevant observations regarding the trade‐offs involved in conservation (e.g., A. N. Sharpley & Smith, 1994) and established routines for process‐based simulation modeling that are still used today (e.g., A. N. Sharpley et al., 1984). Remarkably, Sharpley's research extended to nutrients beyond P, including several well‐cited papers on nitrogen (N) (Rolston et al., 1996) and potassium (K) (A. N. Sharpley, 1989) behavior in soils.

While in Oklahoma, Sharpley formed a variety of close collaborative relationships that extended his relevance to P science, management, and policy. Sharpley contributed to the development of the Environmental Policy Integrated Climate (EPIC) model. Heeding the strong encouragement of Smith, Sharpley collaborated with Jimmy Williams, EPIC's creator, to develop new computational routines describing the fate of P and other major nutrient elements in agricultural soils and in runoff water (A. N. Sharpley & Williams, 1990a, 1990b).

By the 1990s, Sharpley was now recognized as a leader in non‐point source P science. His remarkable career trajectory was disturbed, briefly, with the threatened closure of the USDA‐ARS laboratory in Durant, OK. Wanting to continue conducting P research in an area of the United States more directly impacted by eutrophic impairment, Sharpley explored other options. Enter Harry Pionke, a senior USDA colleague who had worked with Sharpley since his early days in Oklahoma (A. N. Sharpley et al., 1983). Pionke, now the research leader of the USDA‐ARS research unit in central Pennsylvania, recruited Sharpley to run the unit's watershed program.

Sharpley's move to Pennsylvania in 1996 would place him in the midst of concerns over agricultural P and eutrophication of the Chesapeake Bay (Magnien, 2001). There, Sharpley reunited professionally with Pionke, the research leader stretched between laboratory administration and science, and was introduced to Bil Gburek, an agricultural engineer with a long history of research in USDA's Mahantango Creek experimental watershed. Sharpley's arrival served to focus the Pennsylvania team on P fate and transport, applying nested, long‐term observations from the Mahantango Creek experimental watershed to illustrate how the majority of watershed P losses derived from small proportions of the landscape (Pionke et al., 1997). Their elegant explanation and documentation of the “critical source area” hypothesis was reinforced by the region's variable source area hydrology along with surface runoff dominated by saturation‐excess processes (Gburek et al., 1996; Pionke et al., 1995). Combining Sharpley's international perspective on P with Gburek's understanding of hillslope and watershed hydrology, the team brought widespread attention to the critical source area hypothesis and ultimately, global testing of this concept for P in watershed science (McDowell et al., 2024). Over the following decade, Sharpley and colleagues would connect this adaptation of the Pareto Principle to the cost‐effective mitigation of agricultural P runoff, a strategy that predominates to this day (Flaten et al., in press; Liu et al., in press; Osmond et al., in press).

In Pennsylvania, Sharpley emerged as both the dominant scholar of, and collaborative team leader for, the study of P science and management. Humble, often shy, and occasionally surprised by the plaudits he received, Sharpley learned to speak with rare clarity to politicians, farmers, the environmental community, and the general public, at the same time as he honed his writings and presentations to guide fellow researchers and inspire students. Now perched on mountains of data and personal observation, Sharpley helped others to discover elsewhere what he had observed during his own studies (e.g., A. N. Sharpley et al., 2002). Often during discussions, Sharpley would recall his own findings that answered, or were strongly applicable, to emerging questions. So in tune was Sharpley with the science that, at one point, while assisting colleagues in rainfall simulation experiments, he predicted the dissolved P concentration of runoff water within 5 µg L^−1^ or 5 billionths of 1 kg, of the ultimate measurement. During this period, Sharpley provided the scientific underpinnings of the phosphorus index, led large teams to produce coordinated, consensus‐forming research, and spawned widespread replication of his science (Flaten et al., in press; Liu et al., in press; Macrae et al., in press; McDowell et al., 2024; Osmond et al., in press).

In 2006, Sharpley retired from USDA and departed the verdant hills of central Pennsylvania, moving to the limestone bluffs and forests of the Ozark Plateau in Northwest Arkansas. Recruited by the University of Arkansas, in part, to help address long‐standing litigation pitting the State of Oklahoma against the region's poultry industry, located primarily in Arkansas (A. Sharpley et al., 2012). In Arkansas, Sharpley reconnected with long‐standing colleagues from his days in Oklahoma, including Tommy Daniel and Philip Moore, Jr. Sharpley, always the researcher's researcher, took major strides in newly purchased cowboy boots to offer water quality extension and outreach to the region's agricultural industry—all with his signature British accent. Partnering with University of Arkansas’ Mike Daniels and the Arkansas Farm Bureau, Sharpley championed the establishment of Arkansas Discovery Farms, a living laboratory using direct observation to quantify and demonstrate the economic and water quality outcomes of adopting best practices on commercial farming operations (A. Sharpley, Daniels, et al., 2015).

During the last decade of his career, Sharpley's productivity and impact remained on track. Notably, this period brought forays into professional society leadership (President of the Soil Science Society of America), critical analysis of system‐level drivers challenging the sustainable use of P, and local watershed mitigation science. Repeatedly, Sharpley organized teams to reflect upon, and provide vision around, important issues related to agricultural P, generating thought‐provoking works that remain important to P science and management to this day (Dodd & Sharpley, 2016; A. Sharpley et al., 2013; A. N. Sharpley, Bergström, et al., 2015; A. Sharpley et al., 2017). Always an example of the leader as servant, Sharpley was persuaded to wade into local water quality controversies. Sharpley sought to lead consensus around water quality uncertainty concerning a swine operation that had been recently constructed in Arkansas’ Buffalo River Watershed, the United States's first designated national river. Briefly embattled, Sharpley not only persevered, but used the experience to offer new wisdom on science's role in policy and social outcomes.

After a remarkably peripatetic, impactful, and meaningful career, Sharpley finally settled in Northwest Arkansas. On April 5, 2016, Sharpley married Sarah Childers in their home overlooking the shores of Beaver Lake, a focus of Sharpley's past P research studies. In 2021, after leading a major effort to summarize conservation trade‐offs related to P, Sharpley handed off the findings to colleagues to publish (Kleinman et al., 2022) and promptly retired.

## 3 CONNECTING P SCIENCE TO SOIL, WATER, AND NUTRIENT MANAGEMENT OUTCOMES

Sharpley's observational and experimental research, combined with his efforts to explain systems using empirical and theoretical models, advanced the science explaining P behavior and management in agricultural systems. Sharpley's conclusions regarding critical source areas of P loss from agricultural watersheds derived from his long‐standing study of P in settings across the globe. Indeed, as described by McDowell et al. (2024), who revisited the critical source area literature of P, robust evidence continues to confirm the relevance of this concept to P loss from smaller watersheds where targeted agricultural mitigation efforts are most apt to improve measurable water quality outcomes. However, as scale expands in larger watersheds with more complex terrain, more diverse land uses, and greater hydrologic complexity, the role of critical source areas is less clear.

Sharpley's understanding of critical source areas of watershed P supported the development of the P index, a tool for site assessment to aid in targeting remedial management. As described by Osmond et al. (in press), Liu et al. (in press), Flaten et al. (in press), and others, there is a direct connection between Sharpley and the adoption of the P index in the United States and across the world. It was during the development and implementation of the P Index as a watershed management tool that Sharpley honed his skills in scientific consensus building. For instance, the US National P Project, led by Sharpley and funded by USDA, involved broad partnerships across North America, providing a model for coordinated collaborative science that rapidly generated empirical relationships to improve the quantification of P loss potential offered by the P index (Osmond et al., in press). Later, Sharpley would help other regions to improve coordination of P research, such as is documented by Liu et al. (in press) for Norway and Sweden. Even after his retirement, efforts to refine the P index continue to emulate the approaches forged by Sharpley and colleagues (Reitmeier et al., in press).

Sharpley's long career represents a period of massive growth in understanding P behavior and management in the environment. He routinely preceded others in describing the mechanisms controlling P fate. Sharpley's first publications document P loss in tile drainage and the importance of by‐pass flow via biopores (earthworm burrows) in connecting surface sources of P with tile drains (A. N. Sharpley & Syers, 1976). Further, the need to address dissolved P transport in watershed management continues to surprise and challenge many, despite Sharpley's many publications on the topic (Flaten et al., in press; Osmond et al., in press). Reflecting upon Sharpley's long‐standing interests in dissolved P in subsurface drainage, Bailon et al. (in press) synthesize monitoring studies in Illinois of denitrifying bioreactors, a nitrate mitigation practice hypothesized to potentially exacerbate dissolved P losses via reductive dissolution of iron‐bound P compounds (bioreactors generally remove dissolved P, but there is wide variability in efficacy).

Although Sharpley's impact on P science and management is clear, less appreciated are his direct and indirect contributions to broader areas of agricultural nutrient management. Flaten et al. (in press) document the extent to which Sharpley's science underlying management inferences within the P Index is now used in a much broader arena. Indeed, by organizing Sharpley's studies around P application rate, applied P form, P application timing, and P application placement, Flaten et al. (in press) illustrate the origins of the “4 Rs of Nutrient Stewardship,” the widely adopted framework for nutrient management used across government, academia, and industry.

## 4 TRANSDISCIPLINARY SCIENCE AND SYSTEM LEVEL CONSIDERATIONS

The extent of Sharpley's contributions to science, management, and policy arenas reflects his character and his tremendous ability to integrate knowledge, as well as his inclusive and constructive approach to collaboration. Macrae et al. (in press) articulate Sharpley's connection to the collaborative group, SERA‐17 (Southern Extension and Research Activity—Information Exchange Group 17), which served as the forum for the development of many of the concepts, research activities, and syntheses in P science that achieved scientific consensus and public adoption. As described by Macrae et al. (in press), both Sharpley and SERA‐17 embodied key principles of collaboration, motivating large teams of scientists to volunteer, or pursue soft funding, in pursuit of consensus behind science and management. Major achievements and syntheses, such as the US National P Research Project, the P index, and documentation of conservation trade‐offs, can be traced to both Sharpley and SERA‐17. Similar reflections of Sharpley's collaborative leadership, often with little attention brought to himself, are offered by Osmond et al. (in press) and Flaten et al. (in press). Sharpley could lead from in front or from behind (Osmond et al., in press).

It is in this collaborative mode that Sharpley helped to entrench the modern tenets of watershed P science through lucid synthesis of complex themes. Indeed, legacy P provides such an example. While themes of buffering have long been foundational to the study of P cycling in the environment, Sharpley brought together colleagues to extend nascent ideas around “legacy P” (Kleinman et al., 2011; Sharpley et al., 2013) into a mature and inclusive concept that helps to explain the slow‐ or non‐responsiveness of watersheds to remedial efforts. Sharpley and colleagues incorporate explanations of abiotic and biotic P buffering with short‐ and long‐term watershed processes into “the physical cascades and biogeochemical spirals of P along the continuum from soil to rivers and lakes, via surface and subsurface flow pathways” (A. Sharpley et al., 2013).

As an indication of the enduring contribution of the legacy P concept to watershed science, Simpson et al. (in press), Witthaus et al. (2024), and Shober et al. (in press) all describe ongoing efforts to elucidate facets of legacy P described and inspired by Sharpley. Simpson et al. (in press) document the USDA Legacy P Assessment Project, a collaboration of long‐term experimental watersheds with USDA's Conservation Effects Assessment Project. With a retrospective nod, Simpson et al. (in press) interpret the P lability of soils and sediments obtained across seven distinct watersheds, highlighting themes of vertical stratification, soil/sediment P translating to dissolved P loss, and P buffering in legacy P dynamics. Witthaus et al. (2024) consider legacy P in soils of the Lower Mississippi Alluvial Plain and identify the potential for mobilization of geogenic sources under contrasting land uses as a locally important process contributing to water quality concern. Finally, Shober et al. (in press) weigh various definitions of legacy P and propose a definition suitable across disciplines and contexts, continuing Sharpley's efforts to build inclusive and transdisciplinary concepts.

## 5 A RESPECTED LEADER

Sharpley was a quiet leader whose colleagues were drawn to him by respect for his scientific authority, integrity, and collaborative personality. The antithesis of overt ambition, Sharpley was often reluctant to appear in the limelight. When honored for his achievements, Sharpley routinely credited others, only serving to reinforce the admiration of his colleagues, as well as to continue to build their trust and deference (A. Sharpley, 2020). Over time, however, Sharpley learned to embrace his role as a leader, serving as Editor‐in‐Chief and President of the Soil Science Society of America.

As a great scientist, Sharpley mentored many students, postdocs, and peers, some of whom are responsible for these words. Sharpley was always generous with his time, accepting requests for reviewing manuscripts, delivering presentations, and societal service throughout his career. Flaten et al. (in press) highlight Sharpley's incredible networking efforts, as well as his regular support of colleagues, traits that consistently placed him within the center of the nexus for important research initiatives. Further, Sharpley's proper British communication style (spoken and written), genteel nature, and thoughtful reflection engendered widespread admiration. He served as a true model for those who were fortunate enough to have worked with him: A model that will surely endure for generations to come.

## AUTHOR CONTRIBUTIONS

**Peter J. A. Kleinman**: Conceptualization; writing—original draft; writing—review and editing. **Don Flaten**: Conceptualization; writing—original draft; writing—review and editing. **Deanna Osmond**: Conceptualization; writing—original draft; writing—review and editing. **Helen Jarvie**: Conceptualization; writing—review and editing. **Richard McDowell**: Conceptualization; writing—review and editing. **Zachary Simpson**: Conceptualization; writing—review and editing. **Joshua Mott**: Conceptualization; writing—review and editing.

## CONFLICT OF INTEREST STATEMENT

The authors declare no conflicts of interest.
